# Supplementary figures and images for: Single nucleotide polymorphism leads to daptomycin resistance causing amino acid substitution—T345I in MprF of clinically isolated MRSA strains
Source: PLoS One. 2021 Jan 22;16(1):e0245732. doi: 10.1371/journal.pone.0245732 (PMC7822245; doi:10.1371/journal.pone.0245732)

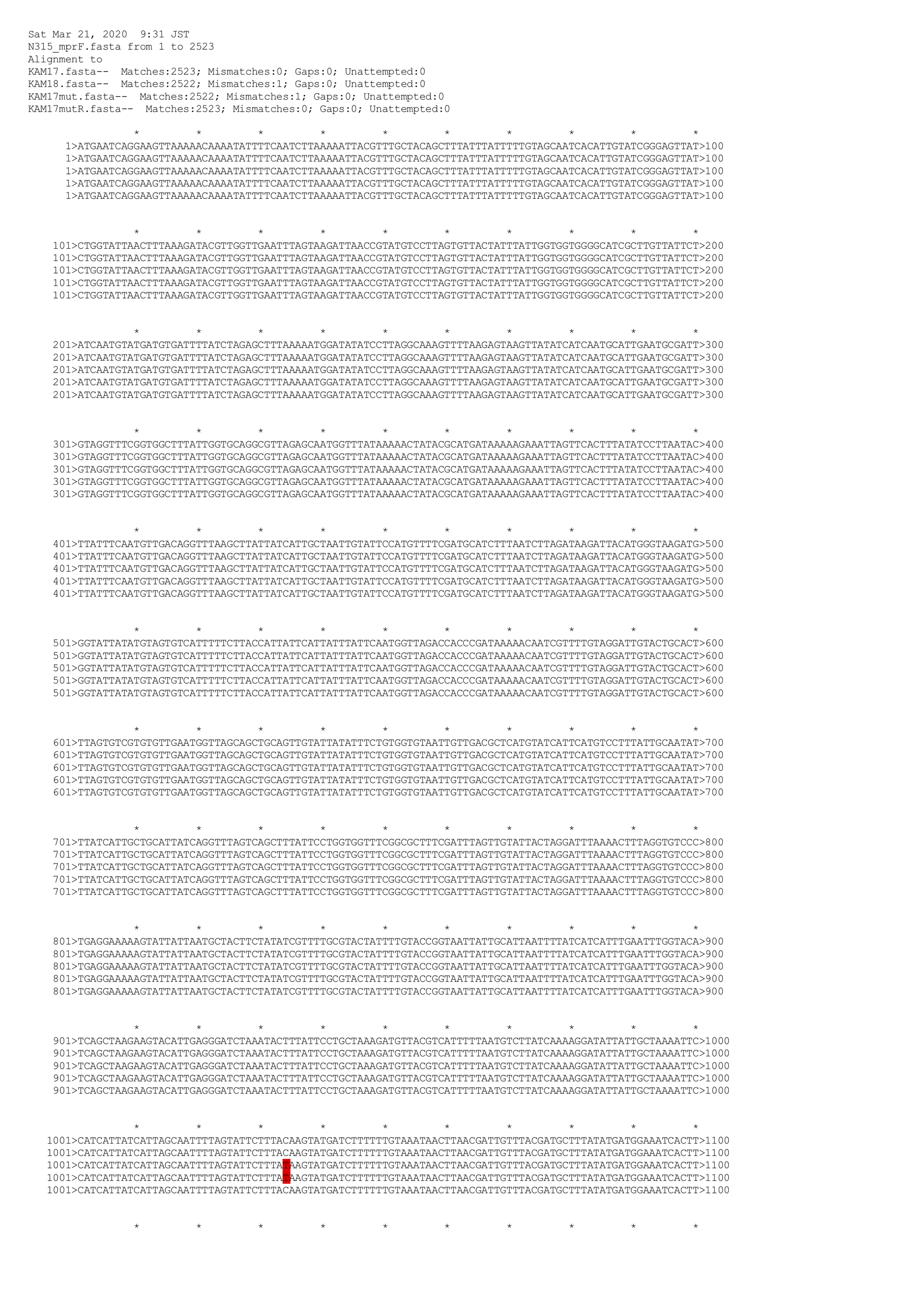

Supplement: S1 Fig — The mprF sequence of four MRSA strains used in this study were aligned with the reference strain MRSA N315. The red background color indicates base mutations. (TIF) [file pone.0245732.s001.tif]

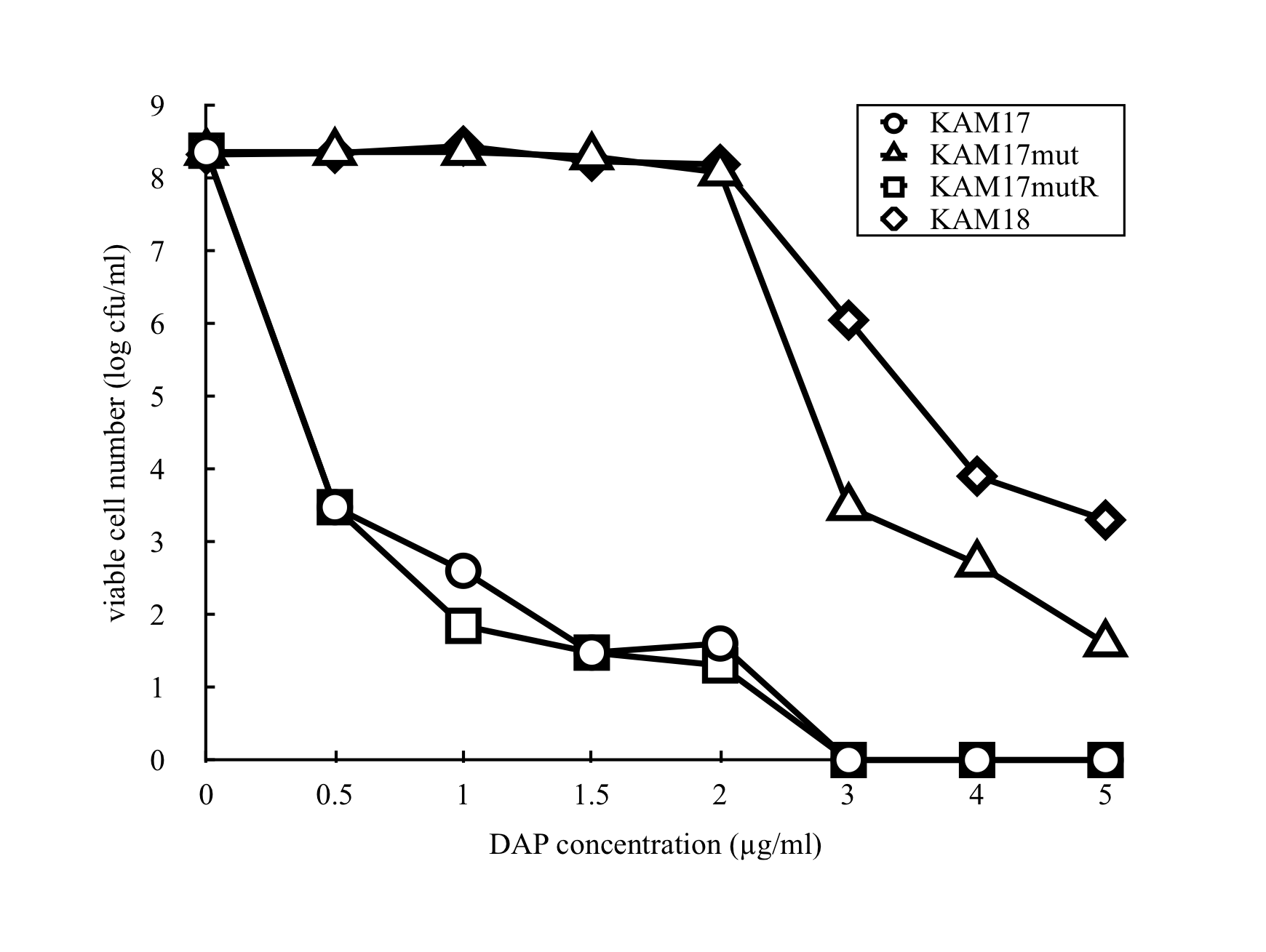

Supplement: S2 Fig — The data represents the additional two independent experiments mentioned in Fig 3. (TIF) [file pone.0245732.s002.tif]

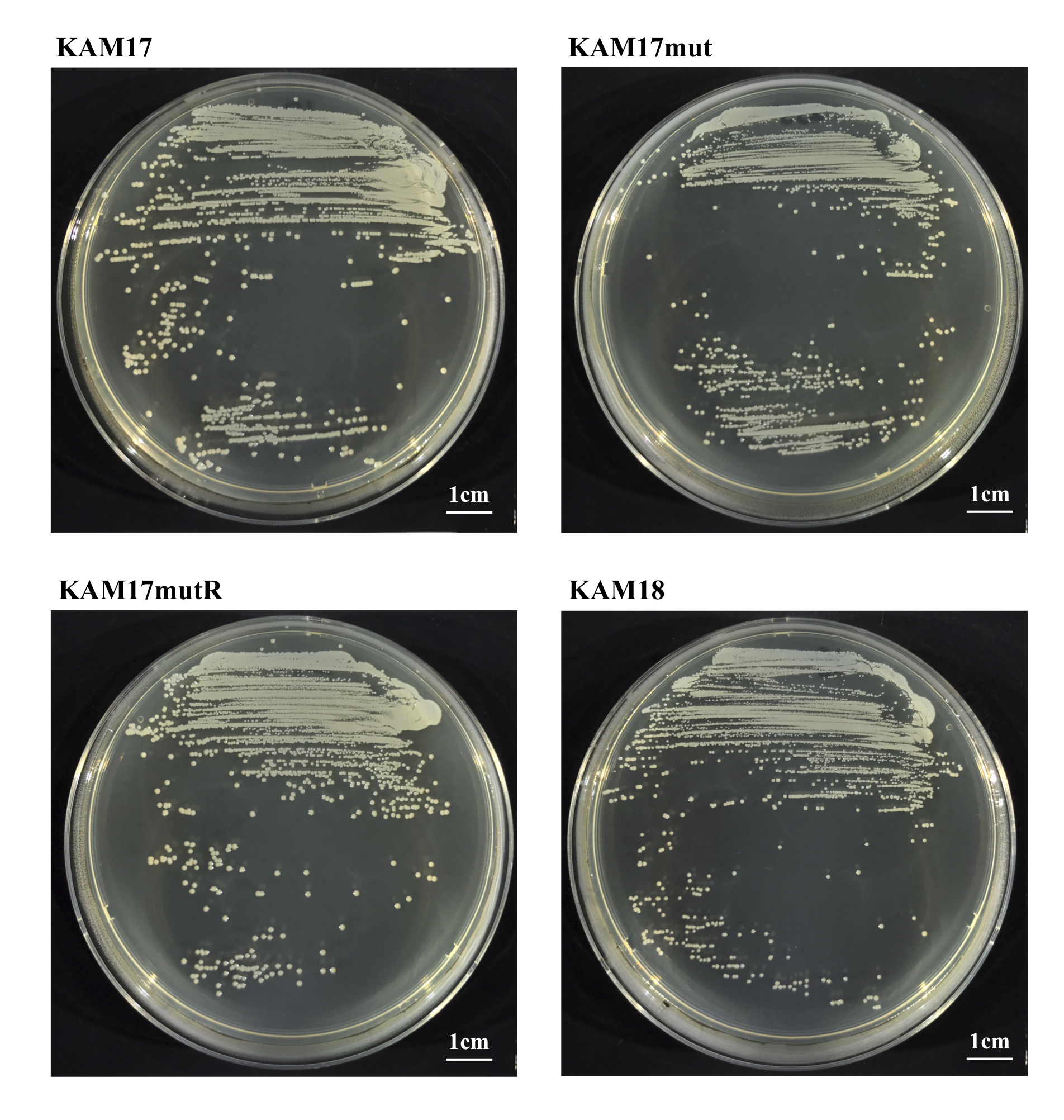

Supplement: S3 Fig — Each strain was cultured on trypticase soy agar for 24 h at 37°C. Size bar = 1 cm. (TIF) [file pone.0245732.s003.tif]

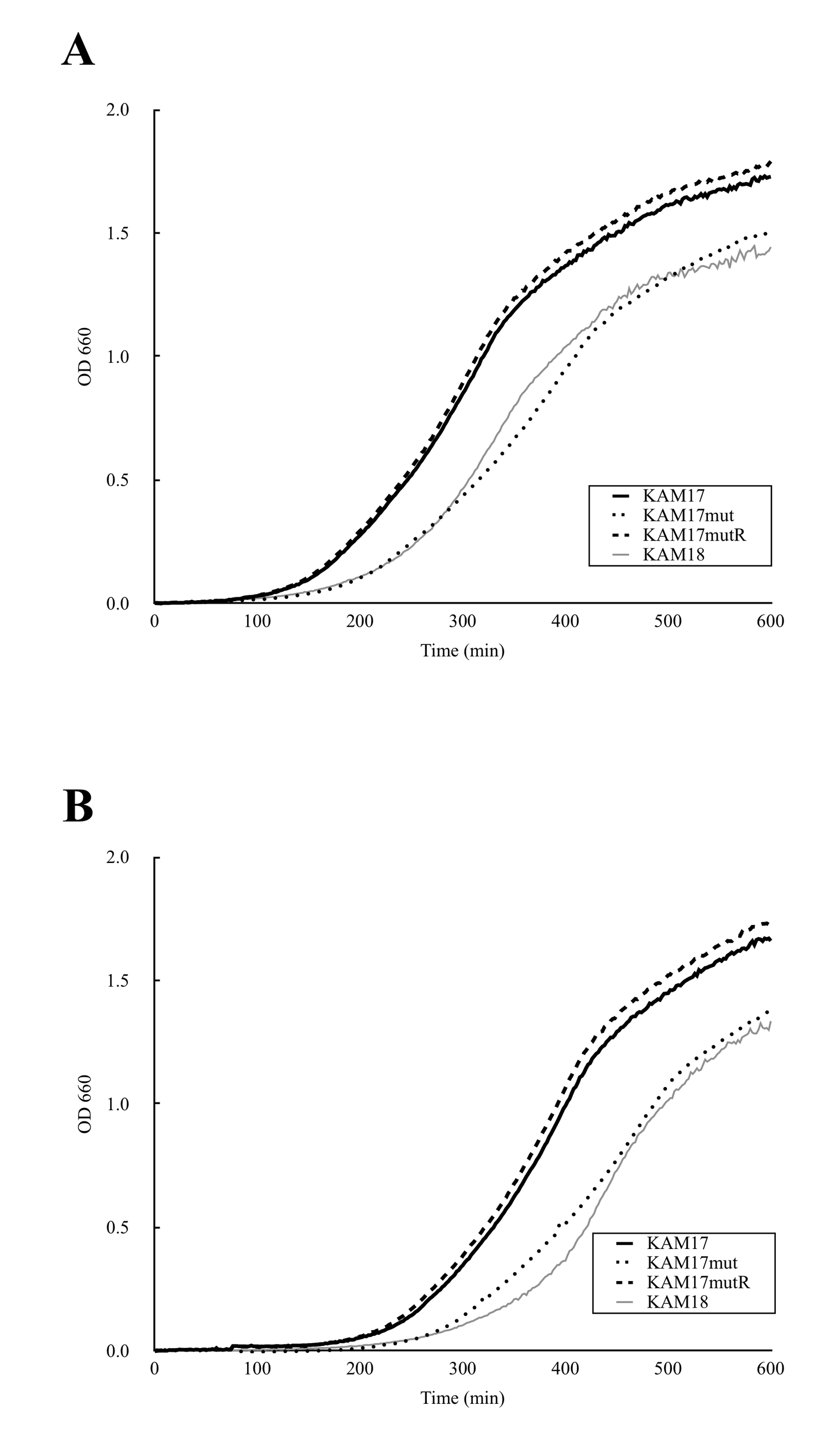

Supplement: S4 Fig — The data represents the three independent experiments mentioned in Fig 5. (TIF) [file pone.0245732.s004.tif]

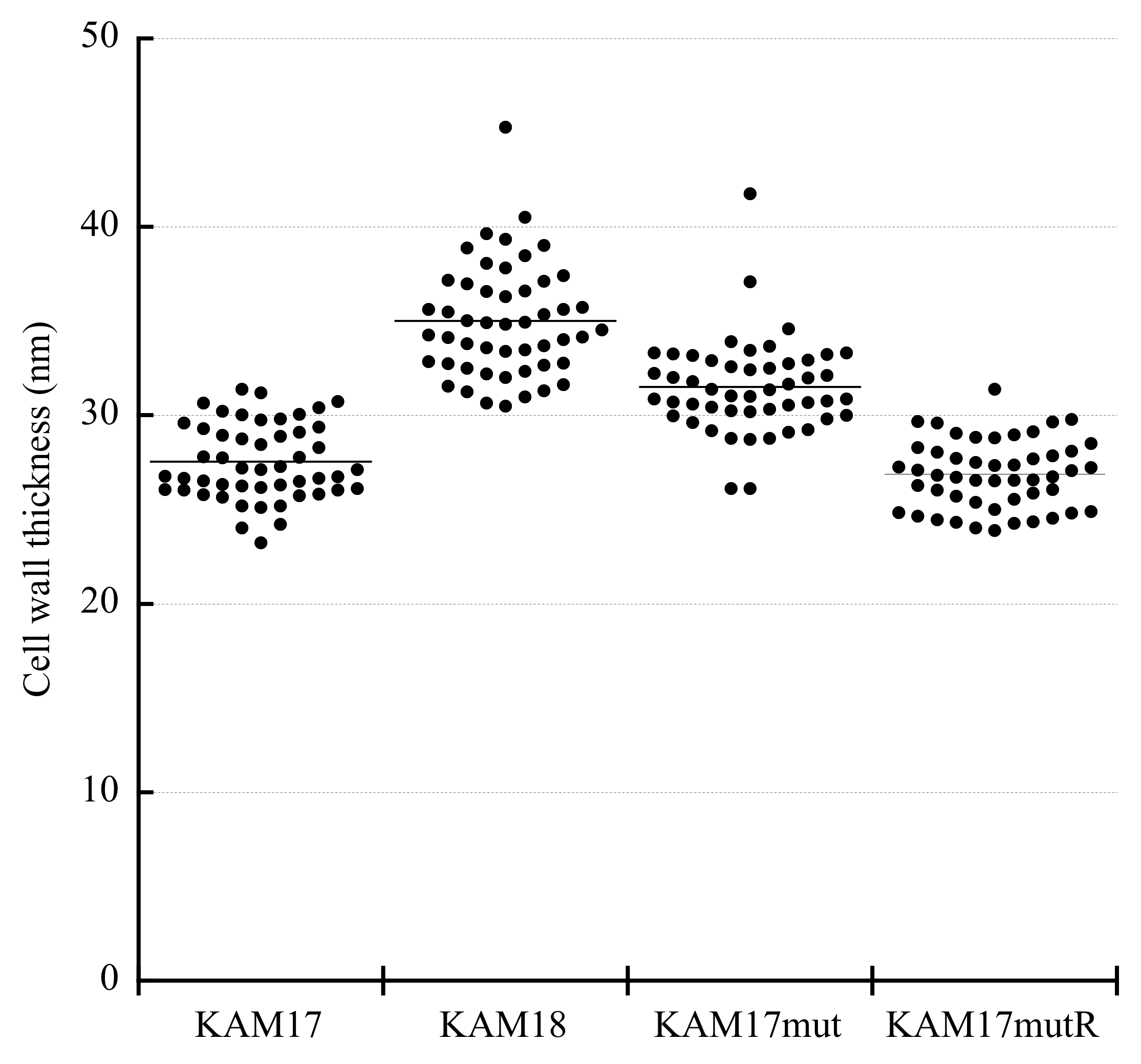

Supplement: S5 Fig — Cell wall thickness was measured at 200,000× magnification. Fifty cells were included in the calculation of cell wall thickness, and results are expressed as dot plot and mean. (TIF) [file pone.0245732.s005.tif]

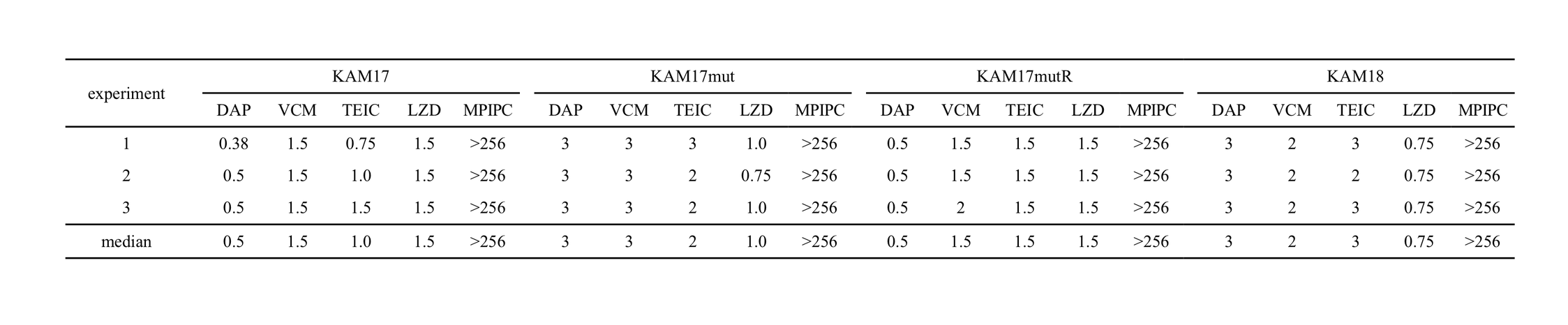

Supplement: S1 Table — (TIF) [file pone.0245732.s006.tif]

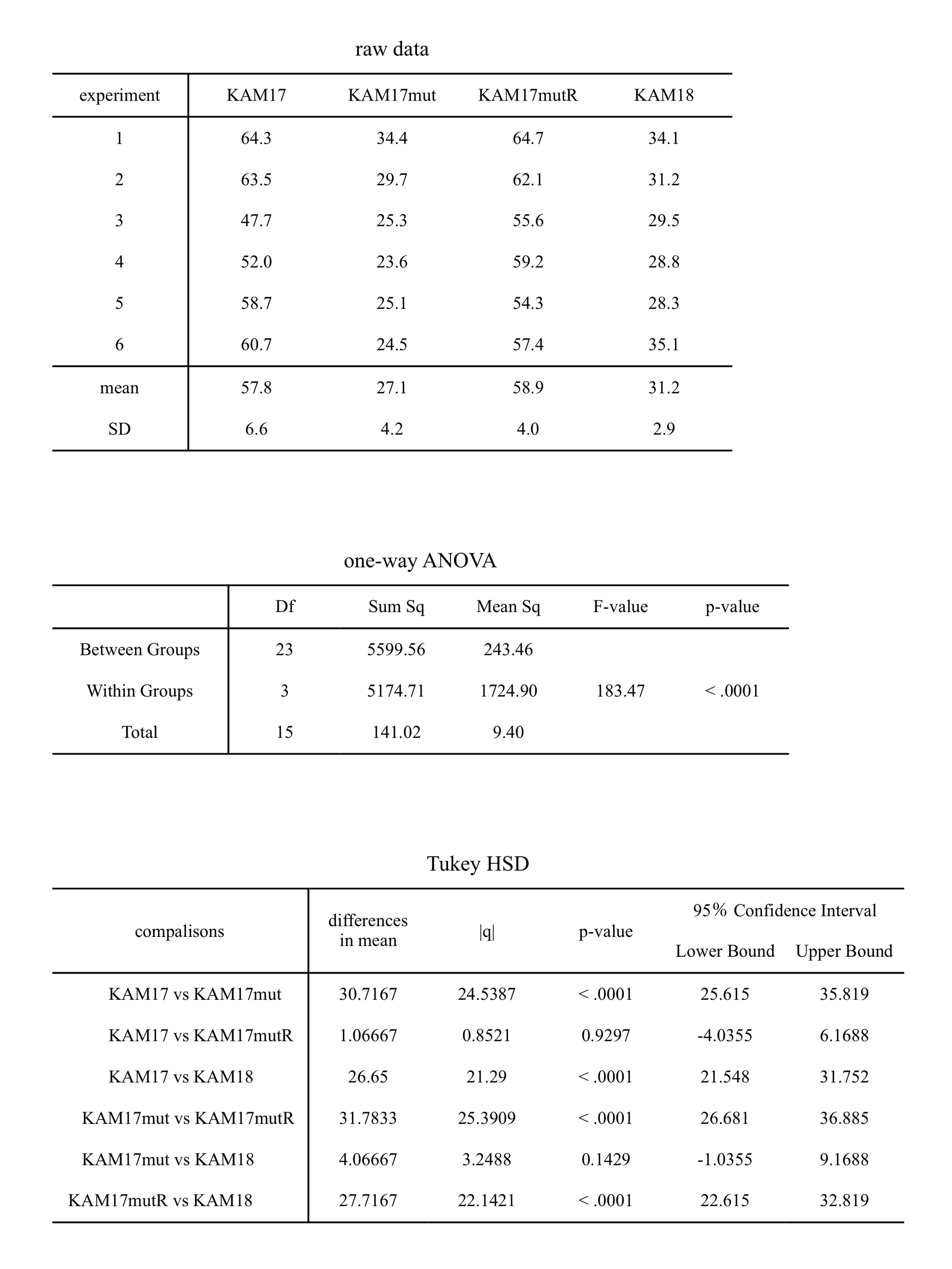

Supplement: S2 Table — (TIF) [file pone.0245732.s007.tif]

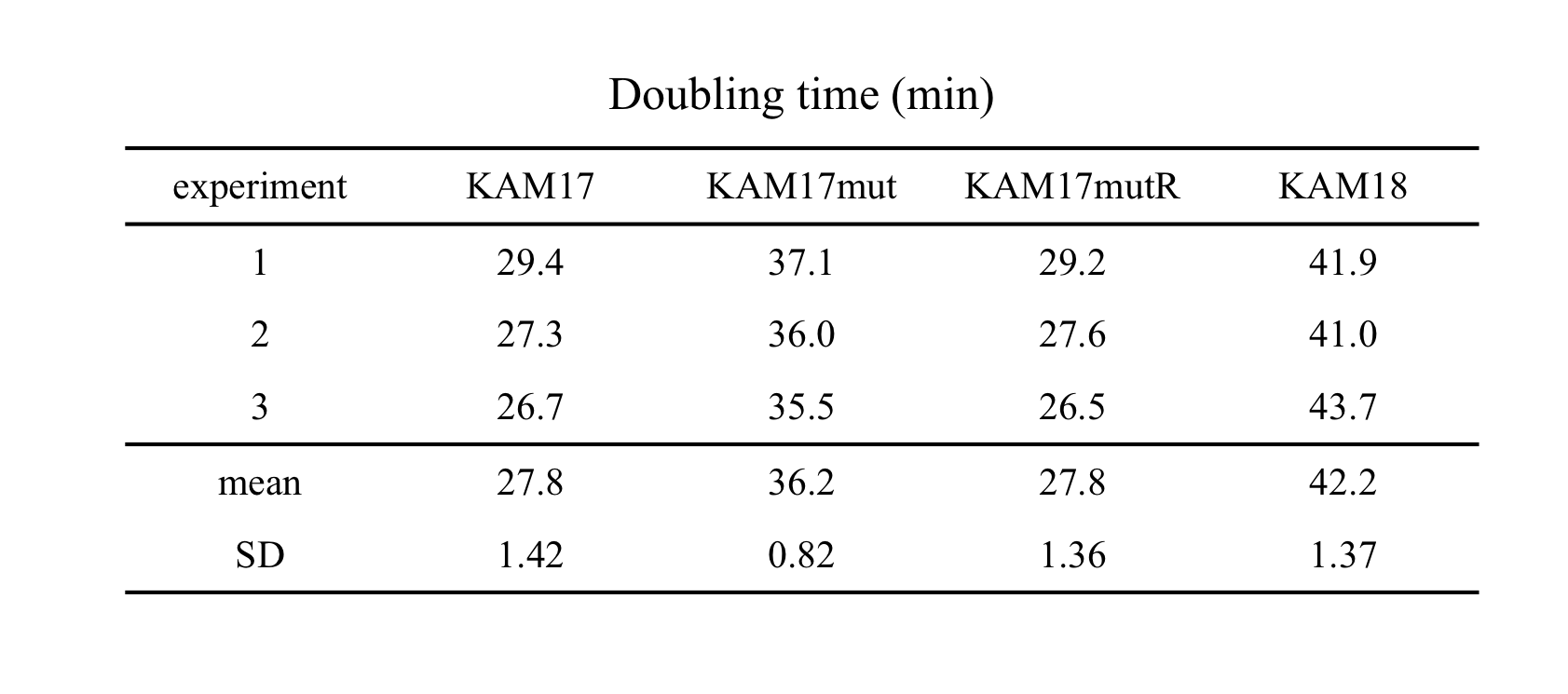

Supplement: S3 Table — (TIF) [file pone.0245732.s008.tif]

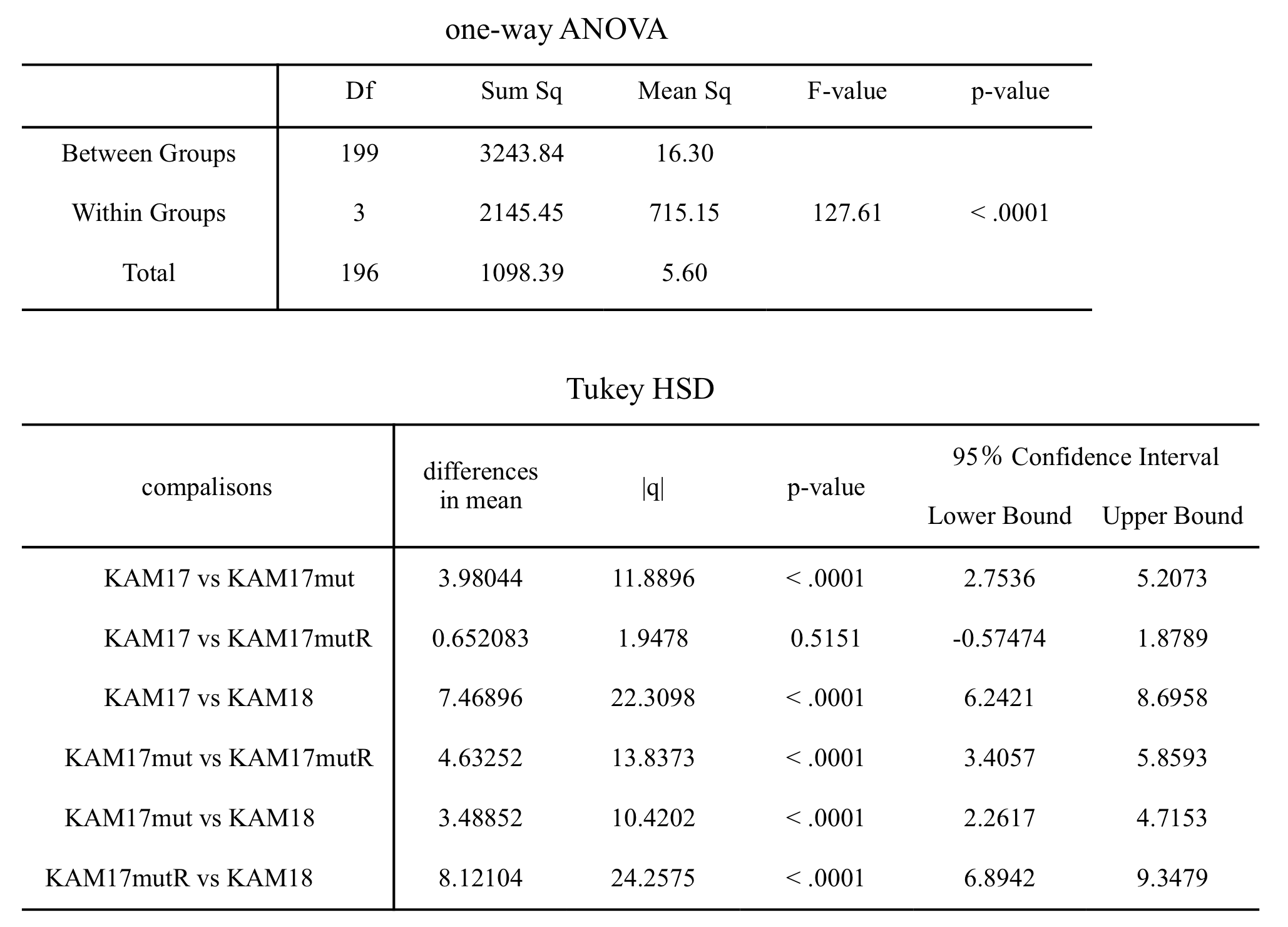

Supplement: S4 Table — (TIF) [file pone.0245732.s009.tif]

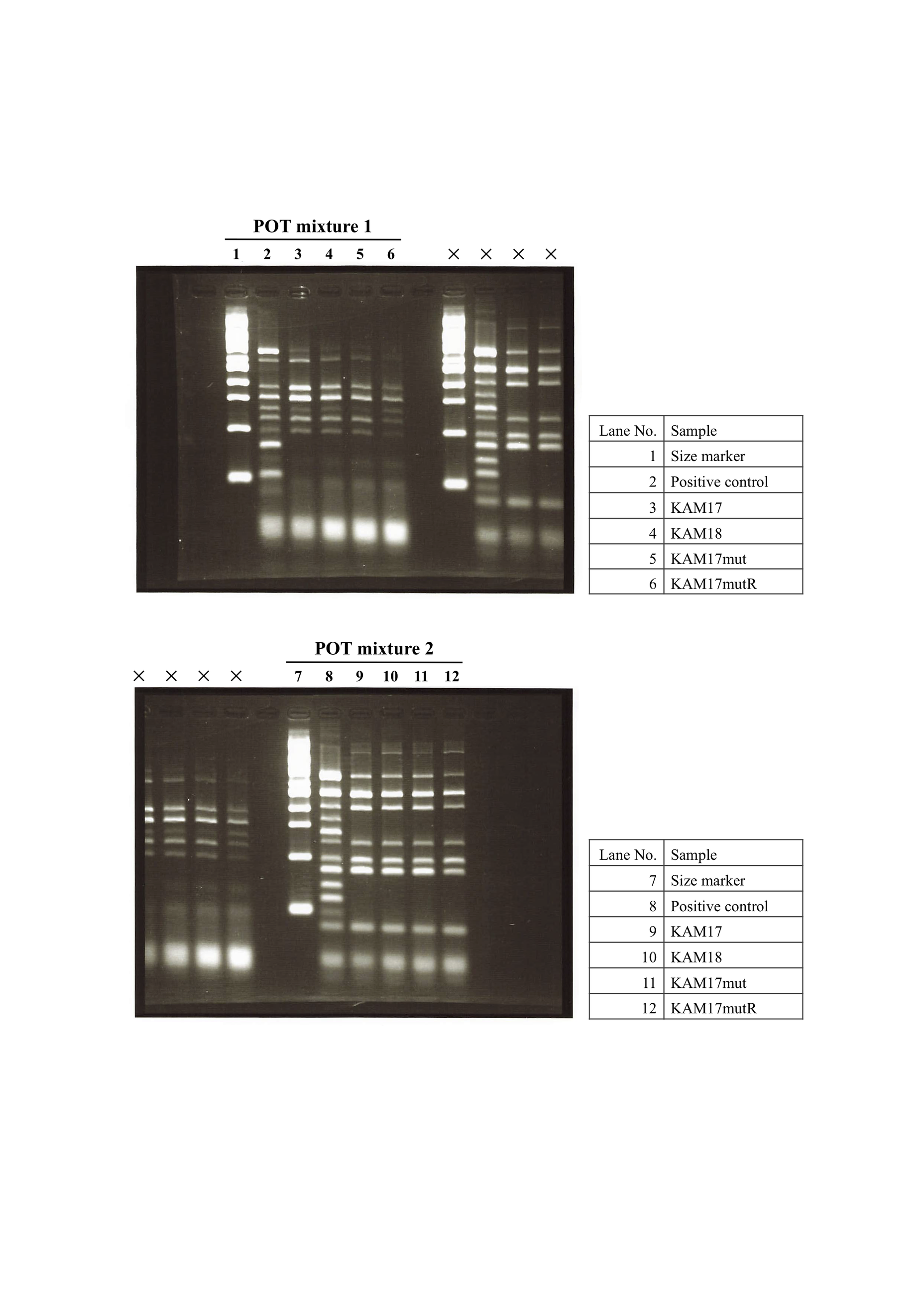

Supplement: S1 Raw images — (TIF) [file pone.0245732.s010.tif]
