# Supplementary material for: Single nucleotide polymorphism leads to daptomycin resistance causing amino acid substitution—T345I in MprF of clinically isolated MRSA strains
Source: PLoS One. 2021 Jan 22;16(1):e0245732. doi: 10.1371/journal.pone.0245732 (PMC7822245; doi:10.1371/journal.pone.0245732)
Supplement: Attachment — Submitted filename: 201226_Response-to-reviewer2.docx [file pone.0245732.s011.docx]

Dear Reviewer #2

Thank you for reviewing our manuscript entitled “Single nucleotide polymorphism leads to daptomycin resistance causing amino acid substitution—T345I in MprF of clinically isolated MRSA strains.” We revised the manuscript according to your comments. The details of the revised sections are as follows.

| Reviewer Comments | Answer to the Reviewer |
| --- | --- |
| Reviewer ＃2 |  |
| 1. Generally, the standard abbreviation of the vancomycin is Vanco or VAN, using VCM sounds odd. | We corrected the abbreviation for vancomycin to “VAN.” |
| 2. I would ask to add the Oxacillin MIC of the studied strains to understand so called “see-saw” phenomenon. | We added the result for oxacillin MIC in each strain. However, the see-saw phenomenon was not observed. |
| 3. In line 47: authors are stating that Vancomycin is the oldest antibiotics which is not true. I would suggest not to overemphasize. | We modified the sentence according to your suggestion. |
| 4. In continuation of line 52; I would suggest to add the couple of sentences of significance of DAP combination therapy approach for the therapeutic purpose particularly for the prevention of DAP-resistance in MRSA. Combination therapy approach is getting popular for treating complicated infections. | We have included a sentence referring to DAP combination therapy. |
| 5. Line 65-66: Please add global references as per the statements. | We have included relevant references. |
| 6. Line 70-72: Among them…………………. Phosphatidylglycerol. Add references. | We have added a relevant reference. |
| 7. The manuscript requires to be reviewed for writing style by English language expert to formulate in better format. | We have had the manuscript edited by a professional English editing service to improve the language, grammar, and overall readability. |
